# Major Radiodiagnostic Imaging in Pregnancy and the Risk of Childhood Malignancy: A Population-Based Cohort Study in Ontario

**DOI:** 10.1371/journal.pmed.1000337

**Published:** 2010-09-07

**Authors:** Joel G. Ray, Michael J. Schull, Marcelo L. Urquia, John J. You, Astrid Guttmann, Marian J. Vermeulen

**Affiliations:** 1 Department of Medicine, St. Michael's Hospital, Toronto, Ontario, Canada; 2 Department of Obstetrics and Gynecology, St. Michael's Hospital, Toronto, Ontario, Canada; 3 Department of Health Policy Management and Evaluation, University of Toronto, Toronto, Ontario, Canada; 4 Institute for Clinical Evaluative Sciences, University of Toronto, Toronto, Ontario, Canada; 5 Department of Medicine (Division of Emergency Medicine), Sunnybrook Sciences Centre, Institute for Clinical Evaluative Sciences, University of Toronto, Toronto, Ontario, Canada; 6 Dalla Lana School of Public Health, University of Toronto, Toronto, Ontario, Canada; 7 Department of Medicine, McMaster University, Hamilton, Ontario, Canada; 8 Department of Clinical Epidemiology & Biostatistics, McMaster University, Hamilton, Ontario, Canada; 9 Department of Paediatrics, The Hospital for Sick Children, Toronto, Ontario, Canada; McGill University, Canada

## Abstract

**Background:**

The association between fetal exposure to major radiodiagnostic testing in pregnancy—computed tomography (CT) and radionuclide imaging—and the risk of childhood cancer is not established.

**Methods and Findings:**

We completed a population-based study of 1.8 million maternal-child pairs in the province of Ontario, from 1991 to 2008. We used Ontario's universal health care–linked administrative databases to identify all term obstetrical deliveries and newborn records, inpatient and outpatient major radiodiagnostic services, as well as all children with a malignancy after birth. There were 5,590 mothers exposed to major radiodiagnostic testing in pregnancy (3.0 per 1,000) and 1,829,927 mothers not exposed. The rate of radiodiagnostic testing increased from 1.1 to 6.3 per 1,000 pregnancies over the study period; about 73% of tests were CT scans. After a median duration of follow-up of 8.9 years, four childhood cancers arose in the exposed group (1.13 per 10,000 person-years) and 2,539 cancers in the unexposed group (1.56 per 10,000 person-years), a crude hazard ratio of 0.69 (95% confidence interval 0.26–1.82). After adjusting for maternal age, income quintile, urban status, and maternal cancer, as well as infant sex, chromosomal or congenital anomalies, and major radiodiagnostic test exposure after birth, the risk was essentially unchanged (hazard ratio 0.68, 95% confidence interval 0.25–1.80).

**Conclusions:**

Although major radiodiagnostic testing is now performed in about 1 in 160 pregnancies in Ontario, the absolute annual risk of childhood malignancy following exposure in utero remains about 1 in 10,000. Since the upper confidence limit of the relative risk of malignancy may be as high as 1.8 times that of an unexposed pregnancy, we cannot exclude the possibility that fetal exposure to CT or radionuclide imaging is carcinogenic.

*Please see later in the article for the Editors' Summary*

## Abstract 2

In a record-linkage study, Joel Ray and colleagues examine the association between diagnostic imaging during pregnancy and later childhood cancers.

## Editors' Summary

**Background:**

In industrialized countries, childhood cancer (any form of cancer in a child aged 14 years or under) remains a major cause of death. With the exception of a few known risk factors, such as acquired genetic predisposition to cancer, which accounts for about 10% of all childhood cancers, the etiology of most childhood cancer remains unknown. There is thought to be an association between exposure to ionizing radiation in pregnancy and the subsequent risk of development of cancer in the exposed mother's child, but the evidence base to support this association is conflicting. For example, studies examining maternal exposure to plain radiographs in pregnancy and subsequent childhood cancer are inconsistent. Furthermore, although their use has dramatically increased over the past two decades, little is known about the cancer risk related to certain types of radiodiagnostic tests, such as CT and radionuclide imaging, both of which expose the fetus to considerably higher doses of radiation than plain radiographs administered at the same anatomical level.

**Why Was This Study Done?:**

Many women could be exposed to major radiodiagnostic tests, such as those used in emergency situations, before they are aware that they are pregnant, as almost 50% of pregnancies are unplanned. This situation means that it is important to determine the subsequent cancer risk to any child exposed to maternal radiodiagnostic tests before birth.

**What Did the Researchers Do and Find?:**

The researchers conducted a retrospective population-based cohort study of women who delivered a live infant in Ontario, Canada between April 1, 1992 and March 31, 2008. The basis of the research was an anonymized database for the whole province of Ontario, where universal health care, including prenatal care and radiodiagnostic testing, is available to all residents. Database characteristics allowed the researchers to link maternal radiation exposure (a major radiodiagnostic test performed on the mother up to one day before her delivery date) in a specific (index) pregnancy to a subsequent malignancy in the child. After birth, maternal-infant pairs were only followed up if the infant was delivered at term, weighed 2,500 g or more, and survived for at least 30 days.

The researchers were able to follow up 1,835,517 maternal-child pairs. The overall rate of exposure to major radiodiagnostic testing in pregnancy was 3.0 per 1,000 and occurred at an estimated mean gestational age of 15.7 weeks. A total of four childhood cancers occurred in the exposed group and 2,539 cancers in the unexposed group corresponding to a crude hazard ratio of 0.69, which did not significantly change after adjustments were made for potential confounding factors, such as maternal age, sex, and the presence of any chromosomal or congenital anomalies in the infant. The overall prevalence of childhood cancer following exposure to CT or radionuclide imaging in pregnancy is under 0.07%, giving an incidence rate of 1.13 per 10,000 person-years.

**What Do These Findings Mean?:**

These findings can help inform clinicians and mothers about the risk of childhood malignancy following major radiodiagnostic testing in pregnancy. The absolute risk appears to be low, while the relative risk is not materially higher than that of unexposed controls. However, as the upper confidence limit of the relative risk of malignancy may be a maximum of 1.8 times that of an unexposed pregnancy, the possibility that fetal exposure to CT or radionuclide imaging is carcinogenic cannot be excluded. Because this finding means that a very slight risk may exist, beta hCG testing should continue to be done in all potentially pregnant women before undergoing major radiodiagnostic testing, and lead apron shielding used in all women of reproductive age, whether or not known to be pregnant. In addition, nonradiation-emitting imaging, such as MRI and ultrasonography, should be considered first, when clinically appropriate. However, some pregnant women will still be faced with the decision to undergo CT or nuclear imaging because the test is clinically warranted. The findings of this study suggest that when clinically indicated, major radiodiagnostic testing in pregnancy should be performed, along with brief counseling to help lessen the anxiety experienced by an expectant mother before and after the birth of her child.

**Additional Information:**

Please access these Web sites via the online version of this summary at http://dx.doi.org/10.1371/journal.pmed.1000337.

- For information for patients and caregivers on radiodiagnostic testing, see The Royal College of Radiologists
- The National Cancer Institute provides information about childhood cancer
- CureSearch for Children's Cancer provides additional information about research into childhood cancer

## Introduction

Cancer remains the second leading cause of hospitalization and death among children aged 14 y and younger in industrialized nations [1]–[3]. Fewer than 10% of childhood cancers are attributable to a genetic predisposition [4]. With the exception of a few known risk factors, including exposure to ionizing radiation in utero and after birth [5],[6], the etiology of most childhood cancer remains unknown.

There has been a substantial increase in the utilization of computed tomography (CT) and radionuclide (nuclear medicine) radiodiagnostic procedures, making them a common source of radiation to patients [7]–[9]. Since these tests are often ordered under emergency circumstances [10], inadvertent exposure in early pregnancy may occur, since half of all pregnancies are unplanned [11].

There may be an association between exposure to ionizing radiation in pregnancy and childhood cancer, yet, the supporting data are somewhat conflicting. For example, studies of cancer incidence following intrauterine exposure to the atomic bomb are inconsistent [12]–[14], as have some [15], but not all [16]–[18] studies examining maternal exposure to plain radiographs in pregnancy. Less is known about the risk related to major radiodiagnostic tests, such as CT and radionuclide imaging, both of which expose the fetus to considerably higher doses of radiation than plain radiographs administered at the same anatomical level [19],[20].

We studied women exposed to major radiodiagnostic tests in pregnancy in an effort to provide clinicians and mothers with better estimates of the risk of pediatric malignancy in their offspring.

## Methods

### Study Design

We completed a retrospective population-based cohort study of women who delivered a liveborn infant in Ontario between April 1, 1992 and March 31, 2008. The study was approved by the Ethics Review Board of Sunnybrook Health Sciences Centre.

### Participants

We included singleton and multifetal pregnancies, and for the latter we included the first infant birth record to avoid multiple counts per pregnancy. We included maternal-infant pairs comprising term liveborn infants who survived more than 30 d after date of birth. For three reasons, we restricted the sample to term infants ≥37 wk gestation, and who weighed at least 2,500 g at birth: First, preterm infants are commonly exposed to radiodiagnostic imaging procedures in hospital [21],[22], which could potentially contaminate the studied effect of prenatal exposure. Second, since term infants represent 94% of all livebirths [23], our findings would remain applicable to most pregnancies. We defined term infants by actual gestational age from 2002 onward, the time in which it was captured in our dataset; before 2002, term status was based on the absence of any diagnostic codes for prematurity according to the International Classification of Diseases, 9th (ICD-9) and 10th (ICD-10CA) revisions (Table S1). Thus, limiting our cohort to term infants enabled us to better estimate the gestational age of fetal exposure to a major radiodiagnostic test prior to the year 2002.

### Exposure and Outcome

The study exposure was CT or radionuclide imaging in an index pregnancy, up to 2 d before the day of delivery (to maximize the likelihood that the fetus was in utero). Prior to the year 2002, gestational age at birth was not recorded in our databases, so the estimated gestational age at exposure was calculated by subtracting 37 wk from the delivery date. This approach has a reported sensitivity of 90% and specificity of 99% [24]. From 2002 onward, we used the recorded gestational age at delivery to determine the timing of exposure to a radiodiagnostic test.

The main study outcome was a confirmed diagnosis of any pediatric malignancy in a child born in the index pregnancy at any time 30 d or more after his/her birth. This 30-d interval was used to exclude infants who died from major morbidity, such as neonatal sepsis or severe anomalies.

### Database Sources

We used anonymized databases for the entire province of Ontario, where universal health care, including prenatal care and radiodiagnostic testing, is available to all residents. Databases were linked using unique encrypted identifiers, which enabled us to link radiation exposure in an index pregnancy to a diagnosed malignancy in a child.

Individual obstetrical deliveries were identified in the Canadian Institute for Health Information Discharge Abstract Database (DAD). The DAD contains age and sex, dates of admission, and up to 16 diagnoses coded by ICD-9 up to March 31, 2002, and up to 25 diagnoses coded by ICD-10-CA thereafter. Up to 2002, inpatient admission records for mothers and newborns are probabilistically linked using delivery/birth dates in the same hospital, same residential postal codes, and diagnostic information to create each mother-child pair. This approach leads to successful matching in 95% of cases. Validation studies of similar maternal-newborn linkages have shown that this approach yields high degrees of successful matching, with a sensitivity of about 95% and a specificity of nearly 90% [25]–[27]. In a Canadian study linking newborn birth records and infant death records, over 99% of records were successfully linked [28]. Starting in the fiscal year 2002/2003, a common identifying number between a delivering mother's chart number and her newborn's chart number permitted a deterministic linkage between the two from 2002 and onward. The DAD was also used to define study covariates (Table S1).

For the study exposure—a major radiodiagnostic test performed on the mother up to one day before her delivery date—we used the Ontario Health Insurance Plan (OHIP) database, which captures all billing information for physician services, including inpatient and outpatient major radiodiagnostic services [29],[30]. In an audit of a representative sample of 11,824 outpatient CT scans and 11,867 outpatient magnetic resonance imaging (MRI) scans identified from 29 randomly selected hospitals across Ontario, 95% of the procedures studied in the chart audit had a corresponding claim in the OHIP database, indicating that these administrative databases have high accuracy in identifying imaging procedures [30]. In the current study, major radiodiagnostic testing included all forms of CT and radionuclide imaging (Table S2). Because the OHIP database does not capture all billings for inpatient radionuclide imaging before 2006, the DAD was used to identify inpatient radionuclide tests performed throughout the study period (Table S2).

For the study outcome—pediatric malignancy—each child record was linked to the Ontario Cancer Registry (OCR), a computerized database of information on all Ontario residents who have been newly diagnosed with cancer [31]. All new cases of cancer, except for nonmelanoma skin cancer, are registered, and about 80% have the tissue pathological diagnosis recorded within the OCR. Validation studies have shown the OCR to be effective in ascertaining cancer cases in the province, with a sensitivity of 98% [32]. Close to 400,000 records are submitted to the OCR annually, and coded using ICD-9 diagnostic codes (Table S1). Incident cases of pediatric malignancy were captured according to a date of diagnosis between May 1, 1991 (a minimum of 30 d after the earliest birth in the study cohort) and March 31, 2009 (a maximum of 18 y after the earliest birth in the cohort).

Mortality data were retrieved from the Registered Persons Database (RPDB), which contains demographic information for all OHIP-eligible individuals. Income quintile and rurality were defined using Statistics Canada census data. The OHIP database was also used to identify outpatient claims for prenatal ultrasonography at any point in pregnancy (Table S2) [33].

### Statistical Analysis

We measured the absolute number of radiodiagnostic tests performed in each livebirth pregnancy in a given year and calculated the annual test rate per 1,000 livebirths, from 1992 to 2008. Changes over time in the rates for radionuclide tests, CT, and both were analyzed using a Cochran-Armitage test for trend.

Time-to-event analyses were conducted using Cox proportional hazards regression models, to derive a hazard ratio (HR) and 95% confidence interval (CI) for pediatric malignancy among children born to mothers exposed versus not exposed to major radiodiagnostic imaging in pregnancy.

The period of observation for each child started 31 d after birth, to ensure that he or she survived long enough to develop, or to be diagnosed with, a malignancy. A child was censored (i.e., determined to have not had a study outcome event) at the point in time in which he or she either reached the end of the period of March 31, 2009 without having experienced a study outcome event or died during the period of study. While emigration from the province could not be ascertained, the annual rate is less than 1% (http://www.statcan.gc.ca/pub/75-001-x/2008110/t/10711/5800473-eng.htm); such persons were classified as being event-free up to March 31, 2009.

The HR was adjusted for maternal age at delivery (in years), income quintile, urban status, and maternal cancer diagnosed in the index pregnancy or up to 6 mo thereafter, as well as infant sex, a chromosomal or congenital anomaly documented at the time of birth or up to 365 d thereafter (present or absent), and exposure to CT or radionuclide imaging (as present or absent) starting 31 d after birth and up to 365 d before either the date that he/she experienced a study outcome event or the date that he/she was censored. The latter considered postnatal exposure to the same ionizing radiation sources as in pregnancy. Information about maternal parity, prior pregnancy loss, smoking, and medication use were not available.

All *p*-values were two-sided, at a significance level of 0.05. All statistical analyses were performed using SAS for UNIX, Version 9.1 (SAS Institute).

## Results

There were 2,018,924 maternal-child pairs initially generated, of which 183,442 (9.1%) were excluded for the following reasons: newborn weight <2,500 g or >6,000 g (*n* = 113,718); preterm delivery <37 wk gestation (*n* = 50,881); multiple births subsequent to the first birth (*n* = 10,116); maternal age <16 y or >50 y (*n* = 4,091); nonresident of Ontario (*n* = 2,310); infant death ≤30 d after birth (*n* = 1,514); stillbirth (*n* = 780); extreme post-term birth >43 wk gestation (*n* = 29); and incorrect coding of birth date (*n* = 3). Among those excluded, the rate of stillbirth was not significantly different among mothers exposed (0.65%) and not exposed (0.84%) to major radiodiagnostic testing in pregnancy. Among all mothers exposed to major radiodiagnostic testing in pregnancy, the characteristics of those who were included and excluded are listed in Table S3.

The final cohort comprised 1,835,517 maternal-child pairs. The demographic characteristics of the 5,590 mothers exposed to major radiodiagnostic testing in pregnancy were generally similar to the unexposed cohort of 1,829,927 women (Table 1). However, the rate of diagnosed cancer during or soon after pregnancy was higher in the exposed mothers (0.63% versus 0.050%), as was the rate of early prenatal ultrasonography (33.0% versus 19.0%).

**Table 1:** Characteristics of mothers and their infants who were and who were not exposed to a major radiodiagnostic testing in pregnancy.

Characteristic^a^	Major Radiodiagnostic Test in Pregnancy	
	Exposed (*n* = 5,590)	Unexposed (*n* = 1,829,927)
*Maternal*		
Mean (SD) age at delivery, y	29.0 (5.7)	29.3 (5.4)
Age at delivery, y		
16–19	168 (3.0)	47,729 (2.6)
20–24	1,013 (18.1)	273,835 (15.0)
25–29	1,667 (29.8)	558,566 (30.5)
30–34	1,662 (29.7)	607,798 (33.2)
35–39	784 (14.0)	265,545 (14.5)
40–44	170 (3.0)	43,674 (2.4)
45–50	8 (0.14)	1,464 (0.080)
Income quintile (Q)		
Q1 (lowest)	1,435 (25.7)	416,705 (22.8)
Q5 (highest)	784 (14.0)	302,374 (16.5)
Urban residence	4,737 (84.7)	1,528,253 (83.5)
Mean (SD) length of stay at delivery hospitalization, d	2.5 (2.2)	2.3 (1.6)
Cancer diagnosis in pregnancy or ≤6 mo after delivery	35 (0.63)	1,004 (0.050)
Prenatal ultrasonography any time in pregnancy	4,956 (88.7)	1,452,376 (79.4)
Prenatal ultrasonography <16 wk gestation	1,845 (33.0)	348,104 (19.0)
*Major radiodiagnostic testing in pregnancy*		
Estimated mean (SD) gestational age at exposure, wk	15.7 (12.8)	—
Estimated gestational age at exposure, wk		
0–14	2,866 (51.3)	—
≥15	2,724 (48.7)	—
Mean (SD) number of major radiodiagnostic tests	1.20 (0.59)	—
Number of major radiodiagnostic tests		
1	4,756 (85.1)	—
≥2	834 (14.9)	—
Major radiodiagnostic test type		—
Radionuclide test^b^	1,527 (27.3)	
CT scan	4,088 (73.1)	—
Anatomical location of the CT scan		—
Extremity or head	2,762 (67.6)	—
Thorax	405 (9.9)	—
Abdomen or spine	448 (11.0)	—
Pelvis	473 (11.6)	—
*Liveborn infant*		
Female sex	2,745 (49.1)	891,969 (48.7)
Mean (SD) gestational age at birth, wk^c^	39.1 (1.2)	39.3 (1.1)
Mean (SD) birthweight, g	3,476 (466)	3,497 (462)
Any chromosomal anomaly	7 (0.13)	2,013 (0.11)
Any congenital anomaly	220 (3.9)	67,284 (3.7)
Any major radiodiagnostic test exposure after birth	244 (4.4)	80,348 (4.4)

a All data are presented as a number (%) unless otherwise indicated.

b Ventilation-perfusion lung scan (89.4%) or thyroid scan (10.6%).

c Determined using data from April 1, 2002 onward.

Among the liveborn infants of mothers exposed and not exposed to a major radiodiagnostic test, just under half were female, and the mean (standard deviation [SD]) gestational ages at birth were 39.1 (1.2) and 39.3 (1.1) wk, respectively (Table 1). The rates of chromosomal (0.13% versus 0.11%) and congenital (3.9% versus 3.7%) anomalies were about the same in both groups, as were the rates of CT or radionuclide imaging after birth (4.4% versus 4.4%).

**Figure 1:**
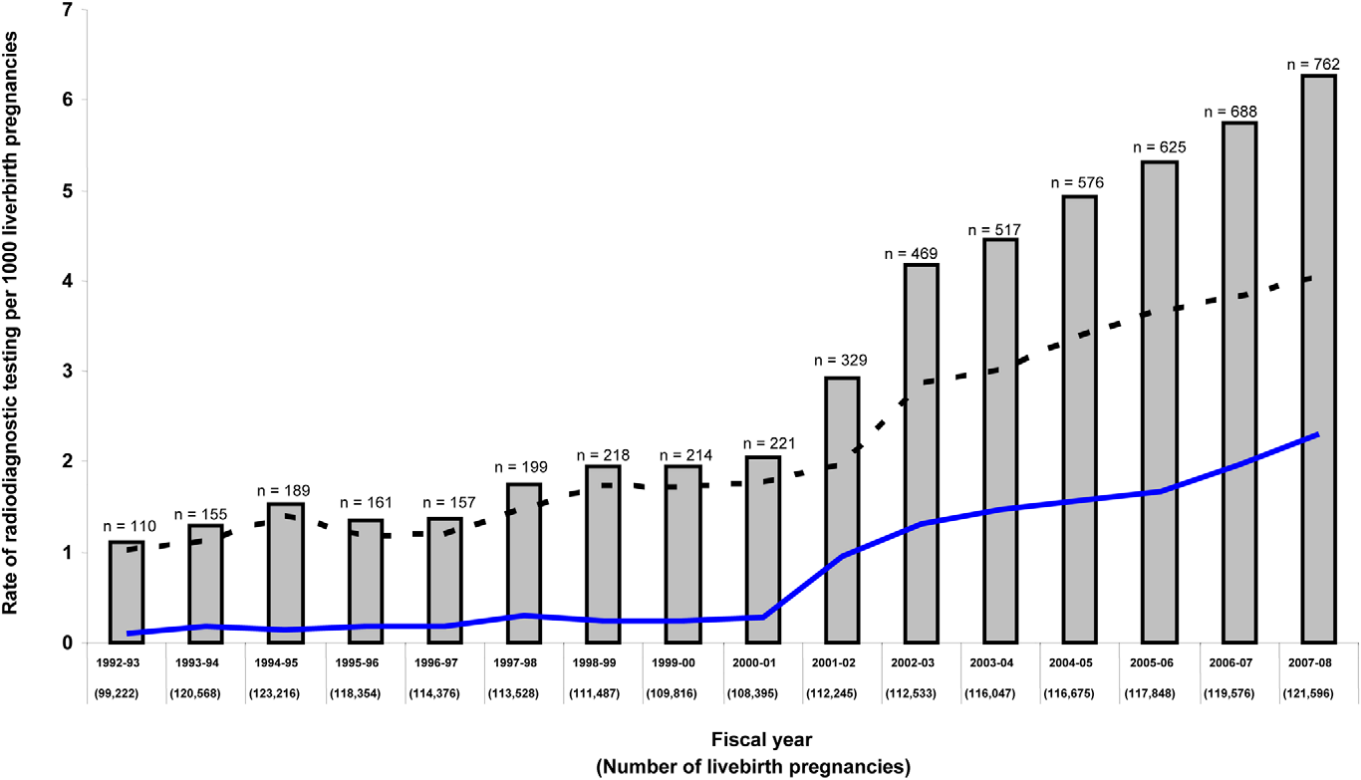
Annual rate of major radiodiagnostic testing in pregnancy in Ontario over time. Data are presented for radionuclide testing (lower solid line), CT scan (upper dashed line), and both (solid bars with total number per year).

The overall rate of exposure to major radiodiagnostic testing in pregnancy was 3.0 per 1,000, which increased significantly over time, peaking at 6.3 per 1,000 livebirth pregnancies in 2008 (trend *p*<0.001) (Figure 1). Exposure to major radiodiagnostic testing in pregnancy occurred at an estimated mean (SD) gestational age of 15.7 (12.8) wk, and about half were completed before 14 wk gestation (Table 1). 15% of women had two or more tests performed in pregnancy. About 73% of all major radiodiagnostic tests were CT scans, of which 68% were of an extremity or head, nearly 10% of the thorax, and 22.6% of the abdomen, spine, or pelvis (Table 1).

There were 35,487 and 16,326,410 persons-years of follow-up in the exposed and unexposed groups, respectively. Among the entire cohort of 1,835,517 children, the median (interquartile range [IQR]) duration of follow-up was 8.91 (4.83–13.00) y. A total of 1,015,789 (55.3%) children were followed to age 8 y, 797,940 (43.5%) to age 10 y, 573,445 (31.2%) to age 12 y, and 341,432 (18.6%) to age 14 y.

**Figure 2:**
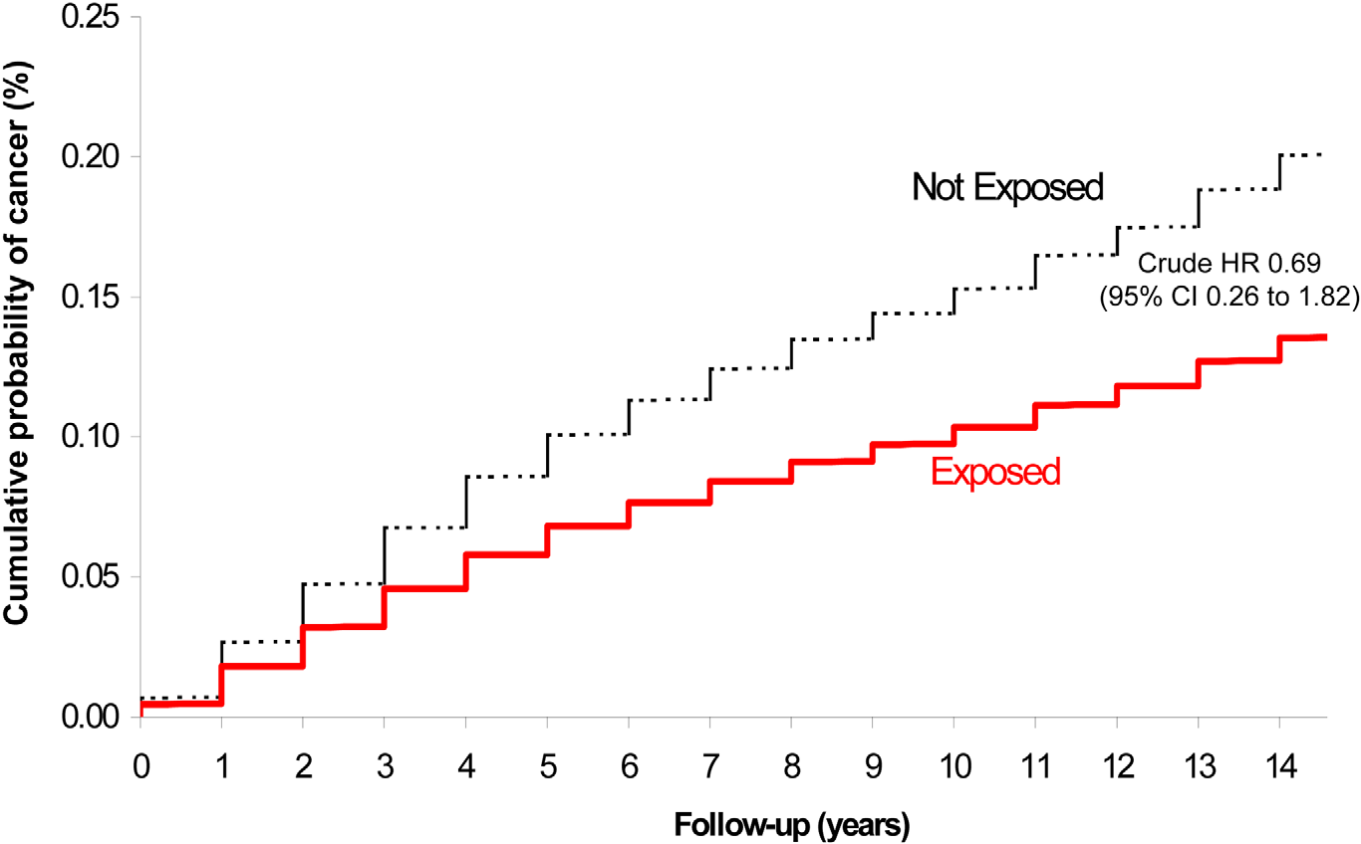
Risk of childhood cancer in the offspring of women exposed (lower solid) and not exposed (upper dashed) to a major radiodiagnostic test in pregnancy.

A total of four childhood cancers occurred in the exposed group (1.13 per 10,000 person-years) and 2,539 cancers in the unexposed group (1.56 per 10,000 person-years), corresponding to a crude HR of 0.69 (95% CI 0.26–1.82) (Figure 2; Table 2). After adjusting for potential confounders, the risk was not significantly higher among exposed versus unexposed maternal-child pairs (HR 0.68, 95% CI 0.25–1.80). Upon adding into the sample previously excluded infants at extremes of gestational age and birthweight, the crude (0.71, 95% CI 0.29–1.70) and adjusted (0.69, 95% CI 0.29–1.66) HR remained unchanged. We could not specify the types of cancers arising in the exposed group because of a strict privacy policy at our institution about the reporting of specific information for cell sizes under the number 5.

**Table 2:** Risk of childhood malignancy in the offspring of women exposed to major radiodiagnostic testing in pregnancy compared to unexposed women.

Major Radiodiagnostic Test Exposure in Pregnancy					
Exposed (*n* = 5,590)		Unexposed (*n* = 1,829,927)		HR (95% CI)
*n* events (%)	Incidence rate per 10,000 person-years	*n* events (%)	Incidence rate per 10,000 person-years	Crude	Adjusted^a^
4 (0.072)	1.13	2,539 (0.14)	1.56	0.69 (0.26–1.82)	0.68 (0.25–1.80)

a Adjusted for maternal age at delivery (continuous in years), income quintile, urban status, and diagnosed maternal cancer in the index pregnancy and up to 6 mo thereafter, as well as infant sex, a chromosomal or congenital anomaly, and exposure to a major radiodiagnostic test exposure after birth.

## Discussion

About 1 in 160 term pregnancies now appear to be exposed to a major radiodiagnostic test. The offspring of women exposed to major radiodiagnostic testing in pregnancy do not appear to be at higher risk of childhood malignancy than the children of unexposed mothers. Moreover, the overall prevalence of pediatric malignancy following exposure to CT or radionuclide imaging in pregnancy was under 0.07%, an incidence rate of 1.13 per 10,000 person-years.

### Study Strengths and Limitations

Among those exposed to major radiodiagnostic testing, totaling nearly 5,600 pregnancies and 35,000 persons-years of follow-up, just four cases of childhood malignancy were identified. The incidence rate of 1.56 cancers per 10,000 person-years, based on 2,539 events arising among 1,829,995 unexposed pregnancies, is highly concordant with population studies from Canada, the United States, Ireland, and Europe [1]–[3]. Post hoc, considering the number of participants enrolled in the current study, at a conventional *p*-value of 0.05, we had just under 20% statistical power to detect the observed difference of 0.40 per 10,000 person-years in the incidence rate of childhood malignancy between exposed and unexposed mothers. To achieve a power of 80%, approximately 60 and 19,613 cancers would be needed in the exposed and unexposed groups, respectively—about eight times higher than the current number of events. That so few cases of childhood malignancy were identified among the exposed is a testament to the difficulty of conducting research in this area, and why previous data may have been lacking. Nonetheless, the wide confidence limits of our relative risk estimates do not exclude the possibility that fetal exposure to CT or radionuclide imaging in pregnancy is carcinogenic, ranging from as low as one-quarter, to as high as 1.8 times, that of an unexposed pregnancy.

We did not include women who had a radiodiagnostic test in a pregnancy that ended in a spontaneous or therapeutic termination before 20-wk gestation. However, this study's focus was on childhood malignancy after 30 d of life, rather than on pregnancy loss, and it is unlikely that including that group of women would have systematically biased the association between the study exposure and outcome. Moreover, the rates of stillbirth after 20 wk among excluded women exposed (0.65%) and not exposed (0.84%) to major radiodiagnostic testing in pregnancy did not differ significantly. By limiting our sample to term deliveries between 37 and 43 wk gestation and restricting the period of exposure up to 2 d before a woman's date of delivery, we improved the accuracy of our determination of the timing of exposure to radiodiagnostic testing. At the same time, our findings may not apply to preterm infants, who both have higher rates of congenital and chromosomal anomalies, as well as major radiodiagnostic testing soon after birth (Table S3) [21],[22].

We did not have on record whether a woman's pregnancy was known at the time of exposure, or if she received lead apron shielding of the abdominal-pelvic area. Assuming that shielding occurred in most cases, as is routine practice, then this may partly explain the low rate of childhood malignancy in the exposed group. Moreover, two-thirds of CT scans were of an extremity or head, with the remaining sites in closer proximity to the fetus, but only 23% were of the abdomen, spine, or pelvis. Hence, much of our CT data reflects “low-risk” scans, in terms of fetal radiation exposure.

We also do not possess information about the generation(s) of CT scans that were used, or the measured dose of ionizing radiation that a woman and her fetus were exposed to. The cited average effective maternal radiation dose of CT of the head or neck is about 2 millisievert (mSv); for a CT of the chest, spine, abdomen, or pelvis it ranges from 6 to 8 mSv [9],[34]; and for a ventilation-perfusion lung scan it is about 2.5 mSv [34]. While these doses are between 10 to 100 times more than that of an equivalent single plain X-ray [34], the effective radiation dose to the fetus remains lower than that to the mother [18],[35]. The rate of major radiodiagnostic testing in pregnancy in our study is certainly lower than when plain X-rays were performed in pregnancy for maternal pelvimetry [36]. In the era of the current study, pelvimetry was not routine, but we would have missed plain X-rays performed for other reasons.

Mothers, as well as clinicians of all types—internists, surgeons, radiologists, obstetricians and midwives, pediatricians, and family and emergency medicine physicians—need data to better estimate the risk of exposure to radiodiagnostic testing in pregnancy. Our findings, though statistically underpowered, inform this process in a manner that has been lacking to date, especially using population-based data over a long period of follow-up, and within a health care system that provides universal coverage of radiological testing and health care.

### Clinical Implications

Our findings can help inform clinicians and mothers about the risk of childhood malignancy following major radiodiagnostic testing in pregnancy. First, the absolute risk appears to be low (about 0.07% or 1 per 10,000 person-years), while the relative risk is not statistically significantly higher than unexposed controls, notwithstanding the wide confidence limits. However potentially small that risk may be, beta hCG testing should continue to be done in all potentially pregnant women before undergoing major radiodiagnostic testing, and lead apron shielding used in all women of reproductive age, whether or not pregnant [6],[37]. Furthermore, nonradiation-emitting imaging (e.g., MRI and ultrasonography) should be considered first, when appropriate.

Some pregnant women will nevertheless be faced with the decision to undergo CT or nuclear imaging because the test is clinically warranted. Unfortunately, some health care providers may be unwilling to counsel a woman about the related fetal risks or may provide misinformation [37],[38]. This issue seems more pressing than ever, given that we and others [9] have noted that CT testing is on the rise, and has become the mainstay for the investigation of pulmonary embolism [39], stroke [40], appendicitis [41], and other common conditions encountered in emergency situations [10]. Both physiological and anatomical changes in pregnancy may obscure the clinical diagnosis of pulmonary embolism [42], for example, such that CT or radionuclide testing may be especially warranted. Delaying the diagnosis of such conditions may postpone therapy, in turn, jeopardizing both mother and child [43].

When indicated, major radiodiagnostic testing in pregnancy should be carried out, along with brief counseling [37]. The latter will hopefully lessen the level of anxiety experienced by an expectant mother (and her family), not only at the time of illness, but after her child is born.

## Supporting Information

Table S1 Diagnostic and procedural codes used to identify the cohort, comorbidity, and outcome features.
(0.03 MB DOC)

Table S2 Codes used to identify inpatient or outpatient major radiodiagnostic imaging (for the study exposure) as well as outpatient prenatal ultrasonography.
(0.04 MB DOC)

Table S3 Characteristics of mothers and their infants who were exposed to a major radiodiagnostic testing in pregnancy and who were included or excluded from the study.
(0.04 MB DOC)

## Abbreviations

CI: confidence interval
HR: hazard ratio
SD: standard deviation
