# Supplementary material for: Major Radiodiagnostic Imaging in Pregnancy and the Risk of Childhood Malignancy: A Population-Based Cohort Study in Ontario
Source: PLoS Med. 2010 Sep 7;7(9):e1000337. doi: 10.1371/journal.pmed.1000337 (PMC2935460; doi:10.1371/journal.pmed.1000337)
Supplement: Table S1 — Diagnostic and procedural codes used to identify the cohort, comorbidity, and outcome features. (0.03 MB DOC) [file pmed.1000337.s001.doc]

Table S1 Diagnostic and procedural codes used to identify the cohort, comorbidity and outcome features

| **Assessment** | **Patient record** | **Disease or procedure** | **Period of assessment** | **ICD-9 [ICD-10-CA] codes** | **OHIP codes** |
| --- | --- | --- | --- | --- | --- |
| ***Cohort inclusion criteria applied to all maternal-child pairs*** | Fetal/infant | - Liveborn, and  - Born ≥ 37 weeks' gestation*  - Survived > 30 days after date of birth | Index birth hospitalization and  ≤ 30 days after birth | - Absence of any code for stillbirth: V320, V350 [P95]  - Absence of any code for prematurity: 765.0, 765.1 [P07.2, P07.3]* | -- |
|  | Maternal | Obstetrical delivery ≥ 20 weeks' gestation of a liveborn infant during the period of observation | Index obstetrical delivery hospitalization | Absence of any code for stillbirth: V271, V273, V274, V276, V277, 656.4 [Z371, Z373, Z374, Z375, Z376, Z377, O364] | -- |
| ***Study exposure*** | Maternal | Radiodiagnostic imaging in pregnancy | Up to 2 days before the index obstetrical delivery date | See Table S2 | See Table S2 |
| ***Study outcomes*** | Infant | - Any malignancy  - Leukemia | > 30 days after index birth hospitalization discharge date | - 140-209.3, 230-234  - 204-208 | -- |
| ***Other variables*** | Fetal/infant | Birthweight (grams) | Index birth hospitalization | -- | -- |
|  | Fetal/infant | Gestational age at delivery* | Index birth hospitalization | -- | -- |
|  | Infant/child | Radiodiagnostic imaging in childhood | 31 days after birth and up to 365 days before either the date that of a study outcome event or the censoring date. | -- | See Table S2 |
|  | Fetal/infant | Multiple gestation | Index birth hospitalization | V31-V37 [O30, O31] | -- |
|  |  | Any congenital or chromosomal anomaly | Index birth hospitalization and ≤ 30 days after birth | 740-759 [Q00-Q99] | -- |
|  | Maternal | Multiple gestation | Index obstetrical delivery hospitalization | 651 [O30, O31, Z37.2-Z37.7,  Z38.3-Z38.8] | -- |

*Prior to the year 2002, ICD-9 and ICD-10-CA codes were used to identify term deliveries. From 2002 onward, actual gestational age was used.

ICD-9 International Classification of Diseases, 9th Revision; ICD-10CA International Classification of Diseases, 10th Revision; OHIP Ontario Health Insurance Plan
