# Supplementary material for: Major Radiodiagnostic Imaging in Pregnancy and the Risk of Childhood Malignancy: A Population-Based Cohort Study in Ontario
Source: PLoS Med. 2010 Sep 7;7(9):e1000337. doi: 10.1371/journal.pmed.1000337 (PMC2935460; doi:10.1371/journal.pmed.1000337)
Supplement: Table S2 — Codes used to identify inpatient or outpatient major radiodiagnostic imaging (for the study exposure) as well as outpatient prenatal ultrasonography. (0.04 MB DOC) [file pmed.1000337.s002.doc]

**Table S**2. Codes used to identify inpatient or outpatient major radiodiagnostic imaging (for the study exposure) as well as outpatient prenatal ultrasonography

| **Type of radiodiagnostic imaging** | **OHIP Professional Fee code** | **CCP [CCI] codes*** |
| --- | --- | --- |
| **CT** |  |  |
| Head | X400, X401, X188C, X402, X405C, X408C | -- |
| Neck | X403, X404C, X124C | -- |
| Thorax | X406, X407C, X125C | -- |
| Abdomen | X409, X410C, X126C | -- |
| Pelvis | X231, X232C, X233C | -- |
| Extremities | X412, X413C, X127C | -- |
| Spine | X415, X416C, X128C, X168, X417 | -- |
| **Radionuclide** |  |  |
| Thyroid | J818, J618, J871, J671, J817, J617, J870, J670 | 06.01 [3.FU.70.CA] |
| Ventilation and/or perfusion lung  scintigraphy | J859, J659, J887, J687, J860, J660 | 06.15 [3.GT.70.CA, 3.GT.70.KC, 3.GT.70.KD, 3.GT.70.KE] |
|  |  |  |
| **Prenatal ultrasonography** |  |  |
| Gestational age < 16 weeks' gestation | J157, J457 | -- |
| Limited for high risk pregnancy or complications of pregnancy | J158, J458 | -- |
| Complete ≥ 16 weeks' gestation | J159, J459 | -- |
| Complete for high risk pregnancy or complications of pregnancy | J160, J460 | -- |

* Indicates coding by CCP (corresponding to ICD-9 years) or CCI (corresponding to ICD-10-CA years)

CCP Canadian Classification of Procedures; CCI Canadian Classification of Interventions; OHIP Ontario Health Insurance Plan
