# Supplementary material for: Major Radiodiagnostic Imaging in Pregnancy and the Risk of Childhood Malignancy: A Population-Based Cohort Study in Ontario
Source: PLoS Med. 2010 Sep 7;7(9):e1000337. doi: 10.1371/journal.pmed.1000337 (PMC2935460; doi:10.1371/journal.pmed.1000337)
Supplement: Table S3 — Characteristics of mothers and their infants who were exposed to a major radiodiagnostic testing in pregnancy and who were included or excluded from the study. (0.04 MB DOC) [file pmed.1000337.s003.doc]

Table S3. Characteristics of mothers and their infants who were exposed to a major radiodiagnostic testing in pregnancy and who were included or excluded from the study

|  | **Major radiodiagnostic test in pregnancy** | |
| --- | --- | --- |
| **Characteristic*** | **Included (n = 5,590)** | **Excluded (n = 1,081)** |
| *Maternal* |  |  |
| Mean (SD) age at delivery, years | 29.0 (5.7) | 29.6 (7.3) |
| Income quintile (Q) |  |  |
| Q1 (lowest) | 1,435 (25.7) | 307 (28.4) |
| Q5 (highest) | 784 (14.0) | 128 (11.8) |
| Urban residence | 4,737 (84.7) | 902 (83.4) |
| Mean (SD) length of stay at delivery, days | 2.5 (2.2) | 11.9 (19.5) |
| Cancer diagnosis in pregnancy or ≤ 6 months after delivery | 35 (0.63) | 46 (4.3) |
| Prenatal ultrasonography any time in pregnancy | 4,956 (88.7) | 909 (84.1) |
| Prenatal ultrasonography < 16 weeks' gestation | 1,845 (33.0) | 302 (27.9) |
|  |  |  |
| *Major radiodiagnostic testing in pregnancy* |  |  |
| Estimated mean (SD) gestational age at exposure, weeks | 15.7 (12.8) | 15.6 (12.3) |
| Estimated mean gestational age at exposure, weeks |  |  |
| 0 to 14 | 2,866 (51.3) | 556 (51.4) |
| ≥ 15 | 2,724 (48.7) | 525 (48.6) |
| Mean (SD) number of major radiodiagnostic tests | | 1.2 (0.59) | | --- | | 1.3 (0.68) |
| Number of major radiodiagnostic tests |  |  |
| 1 | 4,756 (85.1) | 861 (79.7) |
| ≥ 2 | 834 (14.9) | 220 (20.4) |
| Major radiodiagnostic test type |  |  |
| Radionuclide test | | 1,527 (27.3) | | --- | | 224 (20.7) |
| CT scan | | 4,088 (73.1) | | --- | | 863 (79.8) |
| Anatomical location of the CT scan |  |  |
| Extremity or head | 2,762 (67.6) | 553 (64.1) |
| Thorax | 405 (9.9) | 105 (12.1) |
| Abdomen or spine | 448 (11.0) | 93 (10.8) |
| Pelvis | 473 (11.6) | 112 (13.0) |
|  |  |  |
| *Liveborn infant* |  |  |
| Female sex | 2,745 (49.1) | 528 (48.8) |
| Mean (SD) gestational age at birth, weeks** | 39.1 (1.2) | 34.6 (3.0) |
| Mean (SD) birthweight, grams | 3,476 (466) | 2,288 (768) |
| Any chromosomal anomaly | 7 (0.13) | 9 (0.83) |
| Any congenital anomaly | 220 (3.9) | 130 (12.0) |
| Any major radiodiagnostic test exposure after birth | 244 (4.4) | 86 (8.0) |

*All data are presented as a number (%) unless otherwise indicated

**Determined using data from April 1, 2002 onward.
